# Prescription cascades associated with acetylcholinesterase inhibitors use: a high-throughput sequence symmetry analysis

**DOI:** 10.1093/ageing/afag188

**Published:** 2026-06-24

**Authors:** Danielle Newby, Sai Sumedha Bobba, Berta Raventós, Elin J Rowlands, Xihang Chen, Laura Molina-Porcel, Carlen Reyes, Talita Duarte-Salles, Antonella Delmestri, Wai Yi Man, Edward Burn, Marti Catala Sabate, Nicole Pratt, Annika M Jödicke, Daniel Prieto-Alhambra

**Affiliations:** Health Data Sciences (HDS), Translational Sciences, Botnar Research Centre, University of Oxford, Oxford, UK; Health System, University of Alabama at Birmingham Birmingham, Alabama 35294, USA; IDIAP Jordi Gol, Fundació Institut Universitari per a la recerca a l'Atenció Primària de Salut Jordi Gol i Gurina (IDIAPJGol), Barcelona, Spain; Erasmus MC Universitair Medisch Centrum Rotterdam, Department of Medical Informatics, Erasmus University Medical Center, Rotterdam, ZH, The Netherlands; Health Data Sciences (HDS), Translational Sciences, Botnar Research Centre, University of Oxford, Oxford, UK; Health Data Sciences (HDS), Translational Sciences, Botnar Research Centre, University of Oxford, Oxford, UK; Biobanc de l'Hospital Clinic IDIBAPS, Neurological Tissue Bank of the Biobanc-Hospital Clinic-Fundació de Recerca Clínic Barcelona-Institut d’Investigacions Biomèdiques August Pi i Sunyer (FRCB-IDIBAPS), Barcelona, Spain; IDIBAPS–Alzheimer’s disease and other cognitive disorders unit, Neurology Service, Hospital Clínic, Barcelona, Spain; Sardenya Primary Health Care Center, Equip d'Atenció Primària Barcelona Sardenya research unit- Sant Pau Research Institute (EAP Sarden), Barcelona, Spain; IDIAP Jordi Gol, Fundació Institut Universitari per a la recerca a l'Atenció Primària de Salut Jordi Gol i Gurina (IDIAPJGol), Barcelona, Spain; Department of Medical Informatics, Erasmus Medical Center, Rotterdam, Zuid-Holland, The Netherlands; Health Data Sciences (HDS), Translational Sciences, Botnar Research Centre, University of Oxford, Oxford, UK; Health Data Sciences (HDS), Translational Sciences, Botnar Research Centre, University of Oxford, Oxford, UK; Health Data Sciences (HDS), Translational Sciences, Botnar Research Centre, University of Oxford, Oxford, UK; Health Data Sciences (HDS), Translational Sciences, Botnar Research Centre, University of Oxford, Oxford, UK; NHMRC Medicines Intelligence Centre, NHMRC Medicines Intelligence Centre of Research Excellence, Adelaide, South Australia, Australia; Quality Use of Medicines and Pharmacy Research Centre, Clinical and Health Sciences, University of South Australia, Adelaide, South Australia, Australia; Health Data Sciences (HDS), Translational Sciences, Botnar Research Centre, University of Oxford, Oxford, UK; Health Data Sciences (HDS), Translational Sciences, Botnar Research Centre, University of Oxford, Oxford, UK; Erasmus MC Universitair Medisch Centrum Rotterdam, Department of Medical Informatics, Erasmus University Medical Center, Rotterdam, ZH, The Netherlands

**Keywords:** prescription cascade, sequence symmetry analysis, adverse drug events, acetylcholinesterase inhibitors, pharmacovigilance, older people

## Abstract

**Background:**

Acetylcholinesterase inhibitors (AChEIs), are commonly prescribed for dementia and can cause adverse drug events that may lead to new prescriptions, known as prescription cascades. We aimed to identify potential AChEI-induced prescription cascades using high-throughput sequence symmetry analysis (SSA).

**Methods:**

Patients aged ≥18 years with 365 days of prior observation initiating AChEIs (donepezil, rivastigmine, or galantamine) were identified from the Clinical Practice Research Datalink GOLD (2002–2022). We screened 510 drug classes and 1213 individual ingredients initiated within ±180 days of AChEI initiation (365 days in sensitivity analyses). Crude and adjusted sequence ratios (ASRs) were calculated, and positive signals were reviewed for clinical plausibility.

**Results:**

We identified 66 155 AChEI initiators (median age 81 years [IQR 76–85]; 62.8% female). Among ATC classes and individual ingredients, 51 and 46 signals were positive with 28 (55%) and 22 (48%) classified as potential prescription cascades after review. Gastrointestinal drugs showed positive signals including antipropulsives (ASR 1.50 [99% CI 1.28–1.75]), loperamide (ASR 1.52 [1.30–1.77]) and cyclizine (ASR 2.10 [1.72–2.59]). Positive signals were also observed nervous system drugs such as benzodiazepine derivatives (ASR 1.83 [1.55–2.16]) and respiratory drugs including corticosteroids (ASR 1.66 [1.33–2.08]) and glucocorticoids (ASR 1.54 [1.30–1.83]). Most positive signals remained in sensitivity analysis.

**Conclusions:**

These findings suggest potential AChEI-related prescription cascades consistent with gastrointestinal, neuropsychiatric, dermatological and respiratory adverse effects. While findings require further validation, this study demonstrates the utility of high-throughput signal detection to support pharmacovigilance in high-risk populations.

## Graphical Abstract

**Graphical Abstract:**
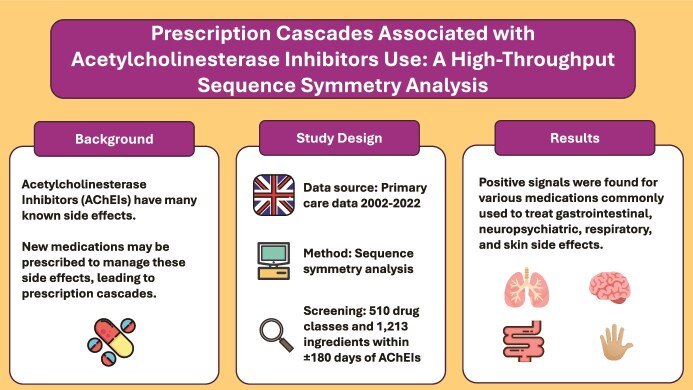

## Key Points

- Prescription cascades occur where a new drug is prescribed to treat adverse effects caused by another drug; Acetylcholinesterase inhibitors (AChEI) may trigger such cascades due to their known adverse drug effects.
- This study identified potential prescription cascades following initiation of AChEI using high-throughput sequence symmetry analysis.
- Identified signals aligned with gastrointestinal, neuropsychiatric, dermatological and respiratory adverse effects, supporting the biological plausibility of these prescription cascades.

## Introduction

Dementia is a growing public health challenge affecting over 57 million people worldwide, with the prevalence projected to grow to 153 million people by 2050 [1]. Acetylcholinesterase inhibitors (AChEIs) (donepezil, rivastigmine and galantamine) enhance neurotransmission by preventing the breakdown of acetylcholine within synapses. They temporarily stabilise cognitive function and delay progression of dementia related to neurodegenerative diseases such as Alzheimer’s disease [2]. However, due to their increased stimulation of the parasympathetic nervous system, AChEIs are associated with a range of adverse drug events (ADEs), including gastrointestinal, urinary, neuropsychiatric and cardiovascular disturbances [3, 4]. Due to their use as first-line treatments for dementia, millions of patients are potentially at risk of prescription cascades [5, 6].

Prescription cascades can occur when an ADE from a prescribed drug is misinterpreted as a new medical condition, prompting the initiation of additional medications to manage the symptoms [7]. This polypharmacy can lead to increased healthcare costs and higher risks of further ADEs, hospitalisation and death [8, 9], particularly in older adults vulnerable to drug-related harms such as those with dementia [10]. Despite the significance of prescription cascades in clinical practice, there is limited research quantifying these events in the context of dementia treatments in real world data.

Sequence Symmetry Analysis (SSA) is a type of self-controlled, case-only screening analysis used for signal detection. It identifies potential signals between two events, typically an index drug and a marker drug by comparing the order of these events within individuals [11, 12]. SSA is particularly well-suited for detecting prescribing patterns in real-world data [13–15], allowing identification of known or unknown ADEs [16]. To date, no studies have systematically applied this method to investigate potential prescription cascades involving AChEIs.

The aim of this study is to identify potential prescription cascades associated with AChEIs using primary care data from the United Kingdom (UK). By employing SSA we seek to enhance understanding of the downstream prescribing consequences of AChEIs and highlight opportunities for mitigating the risks of potential prescription cascades including polypharmacy and drug–drug interactions in this high-risk population.

## Methods

### Study design, setting and data sources

We carried out SSA using routinely collected primary care data from the UK. People with a prescription for AChEIs (donepezil, galantamine and rivastigmine) were identified from the CPRD GOLD database representing ~4.3% of the UK population [17]. CPRD GOLD contains pseudonymized patient-level information on demographics, lifestyle data, clinical diagnoses, prescriptions and preventive care covering >21.3 million patients. CPRD GOLD data was mapped to the Observational Medical Outcomes Partnership (OMOP) Common Data Model [18].

### Study participants

Patients were included if they were 18 years or older with a prescription for the index drugs (AChEIs). From these patients, only those with a marker of interest [Anatomical Therapeutic Chemical (ATC) class or ingredient] within the last 180 days before or after the index initiation in the study period (January 2003 to January 2023) were included. Only those with 365 days of prior history based on the first prescription of the index and markers were included with a 365-washout applied. Patients were excluded if prescription for the index and marker were on the same date.

### Marker definitions

Marker drugs were identified at the class level based on the ATC 4th level for the class level analysis (*n* = 909), and at the ingredient level for the ingredient level analysis (*n* = 31 556). We did not include ATC classes related to vaccines, vitamins, herbal medicines, food, beverages, supplements, tobacco and lab tests. For individual ingredients, we included both pharmacologically active and non-pharmacological agents with the latter may representing markers of clinical events, supportive care, or formulation components.

ATC classes and ingredients with less than 500 patient records were excluded and classes and ingredients representing other anti-dementia drugs. This resulted in 510 ATC classes and 1213 drug ingredients available for analysis. A summary of numbers of excluded classes and drug ingredients can be found in Appendix 1 (Tables S1–S2).

### Statistical methods

We used the R package ‘CohortSymmetry’ [19] to perform SSA for each ATC class and ingredient in a high throughput manner. For each AChEIs (index)-marker pair, we determined the crude sequence ratio (CSR) as the number of patients who initiated the marker class/ingredient after AChEIs initiation divided by the number of patients who initiated the marker before the AChEIs. We required the marker initiation to occur within 180 days prior or following AChEIs initiation with a sensitivity analysis using ±365 days.

To account for changes in the prescribing trends of index and marker over time, the null sequence ratio (NSR) was calculated for each index-marker pair. The NSR is the expected sequence ratio in the absence of any causal relationship between the marker and index drugs, based on population-level prescribing trends [12, 20]. The adjusted sequence ratio (ASR) was calculated by dividing each CSR by the NSR for each pair. An ASRs with a lower 99% confidence interval > 1 were considered a potential positive signal. Adjustment for multiple comparisons were not applied.

To evaluate the performance of SSA before the main study, we tested positive and negative control drug pairs: amiodarone followed by levothyroxine (positive control) and amiodarone followed by allopurinol (negative control). Additionally, we included AChEIs followed by memantine, to reflect a clinically expected treatment sequence in dementia care [21].

Baseline characteristics of patients including age, sex, specific AChEIs medication and a predefined list of comorbidities and medications, were characterised 180 to 1 day prior to AChEIs initiation [22, 23].

All positive signals in the primary analysis were manually reviewed to differentiate potential prescription cascades from false positive signals. False positive signals could be due to various factors such as detection biases, disease progression, comorbidities and reverse causation [14]. Manual review of positive signals first involved assessment of the CSRs, ASRs and NSRs and temporal prescribing patterns. Secondly, literature searches were used to assess evidence for potential prescription cascades and their underlying mechanisms to support signal classification.

All analytical code for this study is publicly available on GitHub (https://github.com/oxford-pharmacoepi/DementiaPSSA). Analyses were conducted using R version 4.2.3.

Individual patient consent was not required, as the CPRD data are de-identified and provided under ethical approval from the UK Health Research Authority (HRA) and the NHS Health and Social Care Research Ethics Committee. The procedures followed were in accordance with the ethical standards of the Helsinki Declaration.

This work was supported by the European Health Data & Evidence Network (EHDEN) has received funding from the Innovative Medicines Initiative 2 (IMI2) Joint Undertaking under grant agreement No. 806968. IMI2 receives support from the European Union’s Horizon 2020 research and innovation programme and the European Federation of Pharmaceutical Industries and Associations (EFPIA). There was partial support from Oxford NIHR Biomedical Research Centre.

## Results

We identified 66 155 initiators of AChEIs who were eligible for inclusion. The baseline characteristics are summarised in Table 1. The majority of AChEIs initiators were female (62.76%), with a median age of 81 years (IQR 76–85) and the highest proportion of initiators aged 80 to 89 years (48.4%). Donepezil was the most prescribed AChEI (73.5%), followed by rivastigmine (17.9%) and galantamine (15.7%).

**Table 1:** Baseline characteristics of acetylcholinesterase inhibitor initiators.

	CPRD GOLD
Number subjects (N)	66 155
**Age**	
Median [Q25 - Q75]	81 [76–85]
Range	19 to 103
**Age group (N %)**	
18 to 49	162 (0.24%)
50 to 59	1029 (1.56%)
60 to 69	5014 (7.58%)
70 to 79	22 083 (33.4%)
80 to 89	31 987 (48.4%)
90+	5880 (8.89%)
**Sex (N %)**	
Female	41 522 (62.8%)
Male	24 633 (37.2%)
**Acetylcholinesterase inhibitors (N %)** ^a^	
Donepezil	48 625 (73.5%)
Rivastigmine	11 811 (17.9%)
Galantamine	10 358 (15.67%)
**Conditions 180 to 1 days prior to index date (N %)**	
Dementia	43 240 (65.4%)
Hypertension	18 572 (28.1%)
Renal impairment	13 080 (19.8%)
UTI	10 940 (16.5%)
Anxiety	10 279 (15.5%)
Depressive disorder	10 245 (15.5%)
Ischemic heart disease	6998 (10.6%)
Hyperlipidemia	6882 (10.4%)
Cerebrovascular disease	6724 (10.2%)
Type 2 Diabetes	6625 (10.0%)
Osteoporosis	5899 (8.92%)
Urinary incontinence	5239 (7.92%)
Atrial fibrillation	4960 (7.50%)
Asthma	4455 (6.73%)
Venous thromboembolism	3304 (4.99%)
COPD	3290 (4.97%)
Stroke	2572 (3.89%)
Myocardial infarction	1910 (2.89%)
GERD	1741 (2.63%)
Pneumonia	1731 (2.62%)
**Medications 180 to 1 days prior to index date (N %)**	
Lipid modifying agents	27 470 (41.5%)
Drugs for acid related disorders	21 983 (33.2%)
Antidepressants	21 864 (33.1%)
Antibacterials systemic	19 700 (29.8%)
Agents for renin ang system	19 360 (29.3%)
Diuretics	17 305 (26.2%)
Antianemic preparations	14 474 (21.9%)
Psycholeptics	14 450 (21.8%)
Opioids	14 423 (21.8%)
Calcium channel blockers	14 132 (21.4%)
Beta blocking agents	12 849 (19.4%)
Antithrombotics	9373 (14.2%)
Drugs for obstructive airway disorders	8972 (13.6%)
Anti-inflammatories and antirheumatics	8538 (12.9%)
Drugs used in diabetes	6453 (9.75%)
Antiepileptics	4232 (6.40%)
Immunosuppressants	631 (0.95%)
Antiemetics	163 (0.25%)

COPD; Chronic obstructive pulmonary disease, GERD; gastroesophageal reflux disease, UTI; urinary tract infection.

^a^Groups are not mutually exclusive.

Within 180 days preceding AChEI initiation, 65.4% of patients had a dementia diagnosis. The most frequent comorbidities were hypertension (28.1%), renal impairment (19.8%) and urinary tract infections (16.5%). Commonly used medications included lipid-modifying agents (41.5%), drugs for acid-related disorders (33.2%) and antidepressants (33.1%).

All study results can be found in a user-friendly interactive web application (https://dpa-pde-oxford.shinyapps.io/dementia_pssa_study/).

Positive and negative controls were in line with expectations: the positive control amiodarone-levothyroxine drug pair showing a positive ASR of 5.33 (99% CI 4.52–6.33), while the negative control amiodarone-allopurinol pair showed a null signal (ASR 0.97 [0.76–1.24]). The study-specific positive control AChEIs-memantine pair showed a positive ASR of 7.18 [5.92–8.79].

**Figure 1:**
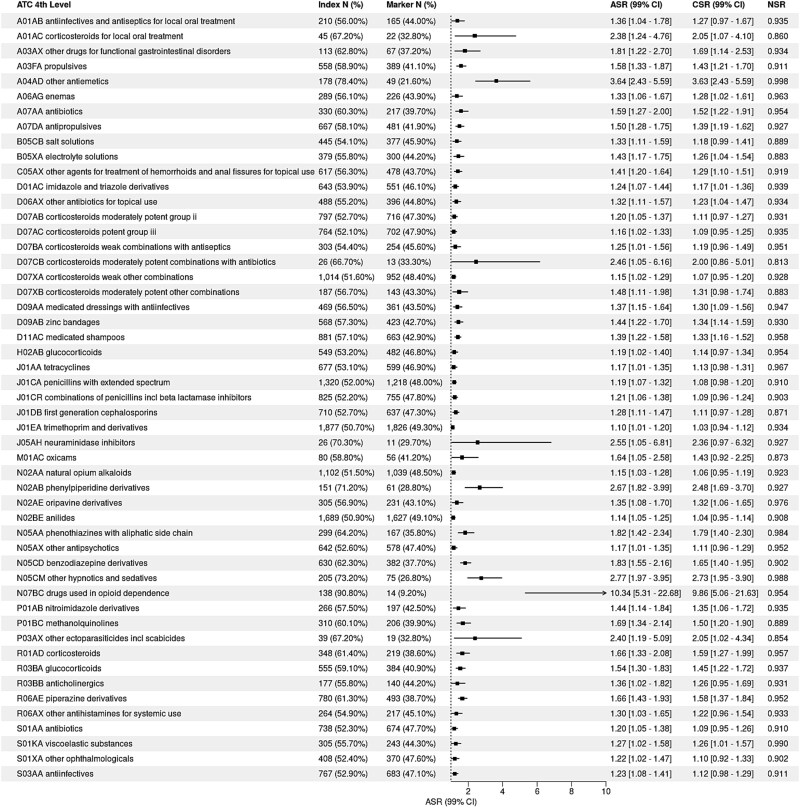
Forest plot of positive signals from high-throughput SSA of acetylcholinesterase inhibitor versus ATC drug classes (4th level) with a 180-day initiation window (ASR: Adjusted sequence ratio, CSR: Crude sequence ratio, NSR: Null sequence ratio).

Among marker ATC drug classes analysed, there were 51 signals identified in the 180-day primary analyses distributed across multiple ATC categories (Figure 1). Notable signals within the ATC alimentary tract and metabolism category included A04AD (other antiemetics) with an ASR of 3.64 (99% CI, 2.43–5.59), and A03FA (propulsives) with an ASR of 1.58 (1.33–1.87). In the nervous system ATC category, N07BC (drugs used in opioid dependence) had an ASR of 9.86 (5.06–21.6) whereas N05CM (other hypnotics and sedatives) had an ASR of 2.77 (1.97–3.95). Additional signals were observed for N02AB (oripavine derivatives 1.35 [1.08–1.70]) and N02AB (phenylpiperidine derivatives 2.67 [1.82–3.99]). Several subgroups in the dermatological ATC class, notably corticosteroids such as D07AB (corticosteroids moderately potent group ii) and D07AC (corticosteroids potent group iii), showed ASRs of 1.20 (1.05–1.37) and 1.16 (1.02–1.33), respectively. Other positive signals were observed for systemic anti-infectives such as J01AA (tetracyclines) and J01CA (penicillins with extended spectrum). ATC classes from the respiratory system also showed positive signals including R01AD (corticosteroids for nasal use) and R03BA (glucocorticoids for obstructive airway diseases) with ASRs of 1.66 (1.33–2.08) and 1.54 (1.30–1.83).

Negative signals (ASR <1) were also observed for some ATC classes (*n* = 16) including drugs for diabetes (A10BA, biguanides; A10BB, sulfonylureas), cardiovascular drugs (thiazides, ACE inhibitors, dihydropyridine derivatives, HMG-CoA reductase inhibitors), and other classes such as antithrombotic agents, antianemic preparations, thyroid therapies, salicylic acid derivatives, certain anti-psychotics (diazepines/oxazepines/thiazepines/oxepines) and antidepressants (e.g. selective serotonin reuptake inhibitors) (Table S3).

Of the 51 positive signals, 42 (82%) remained positive when the index marker initiation window increased to 365 days, with an additional 17 positive signals not in the primary analysis (Figure S1). These additional positive signals included ATC classes for antiemetics (A04AA 1.67 [1.01–2.81]) and drugs for constipation (e.g. A06AA 1.15 [1.04–1.27]). Several nervous system drugs including antiepileptics (N03AG 1.52 [1.14–2.01]), anticholinergic agents (N04AA 1.65 [1.07–2.57]), antipsychotics (N05AD 1.54 [1.29–1.84], N05AL 1.31 [1.06–1.63]), anxiolytics (N05BA) and sedatives (N05CF, N05CH) were also seen as well as iron preparations (B03AB, B03AE), antibacterials (J01DC, J01FA) and nasal preparations (R01AX) (Table S4).

**Figure 2:**
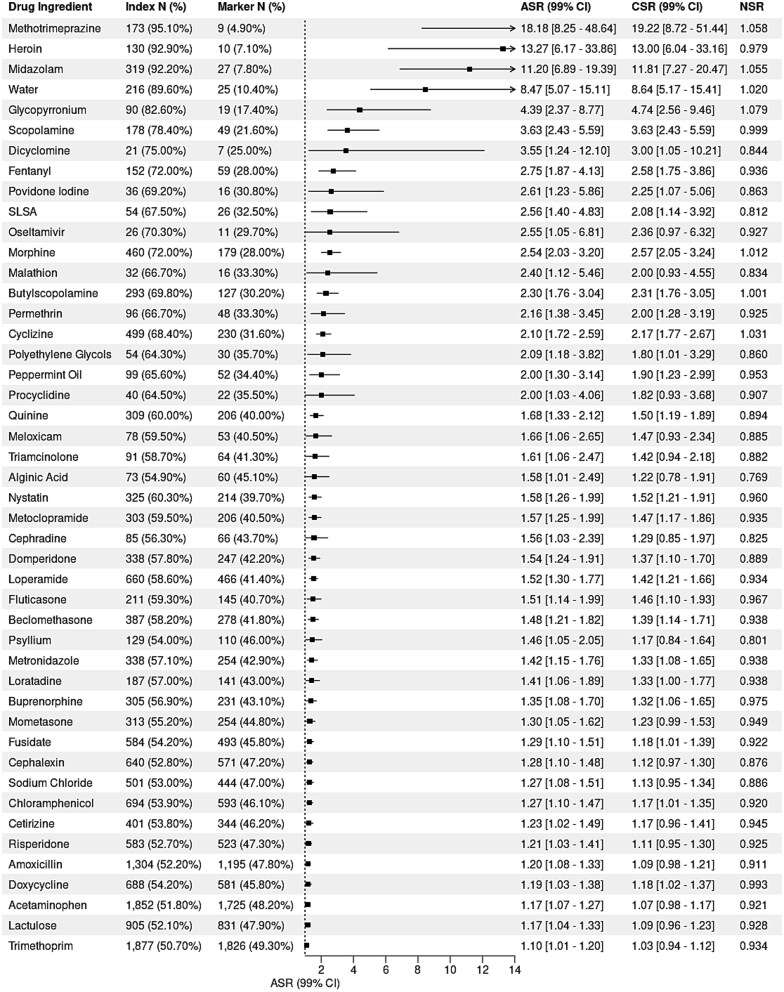
Forest plot of positive signals from high-throughput SSA of acetylcholinesterase inhibitor versus drug ingredients with a 180-day initiation window (ASR: Adjusted sequence ratio, CSR: Crude sequence ratio, NSR: Null sequence ratio).

Among marker ingredients analysed, there were 46 positive signals identified in the 180-day primary analyses (Figure 2). Positive signals spanned ingredients including analgesics, gastrointestinal agents, sedatives, corticosteroids and antipsychotics. Positive signals included opioids such as morphine (ASR 2.54 [2.03–3.20]), fentanyl (2.75 [1.87–4.13]) and buprenorphine (1.35 [1.08–1.70]). Hypnotics and sedatives such as midazolam (11.20 [6.89–19.39]) and methotrimeprazine (18.18 [8.25–48.64]) also were positive. Other ingredients included corticosteroids (beclomethasone 1.48 [1.21–1.82], fluticasone 1.51 [1.14–1.99]), antibiotics (trimethoprim 1.10 [1.01–1.20], metronidazole 1.42 [1.15–1.76], amoxicillin 1.20 [1.08–1.33]) and gastrointestinal agents such as domperidone (1.54 [1.24–1.91]) and loperamide (1.52 [1.30–1.77]). Non-pharmaceutical agents such as water (8.47 [5.07–15.11]) and sodium chloride (1.27 [1.08–1.51]) also showed positive signals.

Negative signals (*n* = 31) were again observed across several therapeutic classes including cardiovascular and anticoagulant agents (e.g. aspirin, amlodipine, apixaban), lipid-lowering therapies (e.g. statins), antidiabetic drugs (e.g. metformin, gliclazide), thyroid and haematological ingredients (e.g. levothyroxine, vitamin B12) and psychotropic medications (e.g. sertraline, quetiapine) (Table S5).

Increasing the initiation window to 365 days, 38 (83%) remained positive with 19 additional positive signals compared to the primary analysis (Figure S2). These additional positive signals included psychotropic and neurological drugs such as amisulpride (ASR 1.30 [1.04–1.64]), trazodone (1.44 [1.23–1.69]), valproate (1.51 [1.14–2.01]), haloperidol (1.53 [1.28–1.83]) and lorazepam (1.58 [1.41–1.77]). Other ingredients included antiemetics (ondansetron), laxatives (sennoside B, docusate), respiratory agents (ipratropium) and analgesics (oxycodone) (Table S6).

Review of positive signals across ATC classes and ingredients determined 28 (55%) and 22 (48%) signals were potential prescription cascades. The review of the positive signals and their prioritisation can be found in Appendix 2 (Table S7 and S8), with additional positive signals from the 365-window sensitivity analysis also provided (Appendix 2, Tables S9 and S10).

## Discussion

This study identified several drug classes and ingredients whose initiation following AChEIs were consistent with possible prescription cascades, where side effects or complications from AChEIs prompted additional medication use. Many positive signals were linked to known ADEs of AChEIs, particularly gastrointestinal, dermatological, neuropsychiatric and respiratory systems.

AChEIs inhibit acetylcholinesterase, preventing the rapid breakdown of acetylcholine in the peripheral nervous system [24]. The accumulation of acetylcholine leads to overstimulation of cholinergic receptions leading to several ADEs including diarrhoea, nausea, dizziness, muscle cramping, tremors, insomnia, urinary incontinence and seizures with evidence showing ADEs are neuropsychiatric, gastrointestinal and cardiovascular in nature [3]. This pharmacological profile provides a plausible biological rationale for several positive signals observed in this work.

Increasing acetylcholine levels cause stimulation of muscarinic receptors in the nasal passages and airways, leading to increased mucus production, rhinorrhea, bronchial secretions and bronchoconstriction [25, 26]; supported by this work and others with signals for medications used to counter these effects such as antihistamines, glucocorticoids and corticosteroids [27]. Gastrointestinal related drugs reflect treatments for common cholinergic side effects such as nausea, vomiting and motility disturbances [28, 29]. Furthermore, signals related to dermatologicals, including topical corticosteroids suggest management of drug-induced skin reactions [28], however, could be due to consequences of ageing and associated care [30]. Psychiatric conditions and treatments are frequently associated with dementia [31]. Positive signals may reflect both side effects of AChEIs [32] and progression of the underlying disease [33]. This interpretation is supported by our sensitivity analysis, which identified additional neurological medications using a wider initiation window. Consistent signals across both windows strengthen confidence in these associations, whereas discrepancies may indicate differences between acute and delayed effects or prescribing practices related to disease progression.

Some signals obtained align with known side effects, such as systemic antibiotics and antiparasitic agents, are more likely attributable to comorbid infections rather than direct prescription cascades. However, there is conflicting evidence regarding AChEIs and infection risk [34, 35]. Furthermore, evidence shows AChEIs are associated with urinary incontinence leading to prescription of antimuscarinic medications, with no positive signals observed in our study. This could reflect under-ascertainment of events, variability in prescribing, awareness of polypharmacy as well as individual AChEIs drug effects. A comparative effectiveness study showed a higher prevalence of antimuscarinic prescriptions comparing donepezil to rivastigmine [36], whereas another study showed no difference between patients with dementia prescribed AChEIs with those not prescribed them [37]. Similarly, a Dutch study identified a positive association between beta-blockers and AChEI [38], whereas our analysis found a negative signal. This difference is likely due to the shorter initiation window of 180 days we used compared to the two years used by the Dutch study. Longer initiation windows can give rise to time-varying confounding, requiring careful consideration of appropriate windows within the disease specific context, with sensitivity analysis required. Differences in population characteristics or prescribing patterns of AChEIs may also explain this discrepancy.

A small number of negative signals were observed in this study. While these should be interpreted cautiously, such findings could suggest either protective potential prescribing patterns, competing risks, or the clinical avoidance of certain medications in patients receiving AChEIs, with clinical guidance recommending reviewing and deprescribing medications to prevent inappropriate prescription cascades in older populations [39].

Beyond acting as proxies for ADEs, potential prescription cascades are also clinically important. The initiation of additional treatments to manage side effects contributes to polypharmacy, increasing the risk of drug–drug interactions, ADEs and treatment burden [9]. Drugs such as benzodiazepines, which may increase sedation and fall risk [40], gastrointestinal agents such as domperidone, which are associated with QT prolongation [41] showed positive signals and illustrate how potential prescription cascades may contribute to polypharmacy and drug–drug interactions following AChEI initiation. In older adults with dementia, such prescription cascades may further increase the risk of ADEs and contribute to cognitive decline [42].

Strengths of this study include the use of a large, comprehensive electronic health records database that enabled real-world pharmacoepidemiologic signal detection across a large population. The use of SSA reduced bias from time-invariant confounders and accounted for certain forms of time-varying confounding via adjustment of potential prescribing trends. Analyses were conducted at both the drug class (ATC level) and individual ingredient level, allowing identification of potential patterns that may be obscured at the class level and decreased power at the ingredient level offering complementary insights.

However, our study has some limitations. SSA is a signal detection method used to detect temporal asymmetry; therefore, it cannot be used to establish causality, with further validation of positive signals required using more robust epidemiological methods [43]. Given the high-throughput nature and the absence of formal adjustment for multiple comparisons, there is an increased risk of false positive findings. Although we used 99% confidence intervals, some observed associations may still have occurred by chance. Furthermore, some signals may reflect disease progression and/or treatment of comorbidities rather than prescription cascades. For example, increased use of antipsychotics or sedatives could be driven by worsening dementia symptoms, which may occur following the initiation of AChEIs [44, 45]. Prescription records in primary care identify if a medication was prescribed, but they do not confirm whether the patient took the drug or adhered to the treatment regimen. Additionally, as AChEIs are often initiated in specialist care before appearing in primary-care records, early ADEs or prescribing cascades that may occur might be missed leading to misclassification and attenuation of signals. We did not restrict our population to individuals over 65 years of age, based on the assumption that the biological processes underlying the development of side effects from AChEIs would not differ by age with most of the population in this study being older any attenuation due to including younger individuals is expected to be minimal.

Our analysis was limited to prescribed medications and did not capture over-the-counter treatments/supplements with some ADEs of AChEIs managed with these treatments, which may underestimate the occurrence of prescribing cascades. Although AChEIs are primarily for dementia, some off-label indications may occur, potentially introducing heterogeneity in prescribing patterns observed following treatment initiation. Finally, some positive signals may reflect confounding by indication, reverse causation, treatment of comorbidities and/or observational period imbalance.

## Conclusion

In summary, this study identified potential prescription cascades associated with the initiation of AChEIs. Many associations corresponded with known ADEs, supporting the biological plausibility of these findings, while others may reflect disease progression or indirect prescribing behaviours. These findings demonstrate the value of high-throughput signal detection for identifying potential prescription cascades to help inform pharmacovigilance in high-risk populations. Further studies using complementary epidemiologic designs are warranted to confirm and validate these findings.

## Supplementary Material

aa-26-0309-File005_afag188

aa-26-0309-File006_afag188

## Data Availability

This study is based in part on data from the Clinical Practice Research Datalink (CPRD) obtained under licence from the UK Medicines and Healthcare products Regulatory Agency. The data are provided by patients and collected by the NHS as part of their care and support. The interpretation and conclusions contained in this study are those of the author/s alone. Patient level data used in this study were obtained through an approved application(CPRD application number 23_003334) and are only available following an approval process to safeguard the confidentiality of patient data. Details on how to apply for data access can be found at https://cprd.com/data-access.
